# Evaluation of Mobile Apps Targeted at Patients With Spondyloarthritis for Disease Monitoring: Systematic App Search

**DOI:** 10.2196/14753

**Published:** 2019-10-28

**Authors:** Yu Heng Kwan, Wei Jie Ong, Mengfei Xiong, Ying Ying Leung, Jie Kie Phang, Charmaine Tze May Wang, Warren Fong

**Affiliations:** 1 Program in Health Services and Systems Research Duke-NUS Medical School Singapore Singapore; 2 Department of Rheumatology and Immunology Singapore General Hospital Singapore Singapore; 3 Duke-NUS Medical School Singapore Singapore; 4 Department of Family Medicine SingHealth Singapore Singapore; 5 Yong Loo Lin School of Medicine National University of Singapore Singapore Singapore

**Keywords:** spondylarthropathies, mobile apps, ankylosing spondylitis, psoriatic arthritis

## Abstract

**Background:**

There are many apps developed for patients with spondyloarthritis in the market, but their purpose and quality are not objectively evaluated.

**Objective:**

The objective of this study was to identify and evaluate existing publicly available, high-quality apps that use validated measurement instruments for monitoring spondyloarthritis disease activity.

**Methods:**

We conducted a review of apps available on the Apple App Store and the Google Play Store based on a combination of keywords and inclusion and exclusion criteria. Validated disease activity measurement instruments were identified. Data regarding app characteristics, including the presence of validated disease activity measurement, were extracted. The Mobile App Rating Scale (MARS) was used to review the apps for user experience.

**Results:**

A total of 1253 apps were identified in the app stores, and 5 apps met the criteria and were further analyzed. Moreover, 2 apps (MySpA and Group for Research and Assessment of Psoriasis and Psoriatic Arthritis App) contained some of the validated disease activity monitoring instruments for specific spondyloarthritis subtypes. These 2 apps were also rated good on the MARS (with total mean scores ≥4 out of 5), whereas the other apps scored poorly in comparison.

**Conclusions:**

There are 2 high-quality spondyloarthritis disease activity monitoring apps publicly available, but they only target 2 spondyloarthritis subtypes—ankylosing spondylitis and psoriatic arthritis. There is a lack of high-quality apps that can measure disease activity for other spondyloarthritis subtypes, and no app that consolidates all validated disease activity instruments across subtypes was available.

## Introduction

### Background

Spondyloarthritis (SpA) is a heterogeneous group of chronic inflammatory diseases with interrelated clinical features and genetic linkages and includes ankylosing spondylitis (AS), psoriatic arthritis (PsA), inflammatory bowel disease–associated arthritis, reactive arthritis, and undifferentiated SpA [1]. These phenotypically diverse and unpredictable diseases are associated with decreased performance of activities of daily living, quality of life, and work productivity [2]. One of the goals for SpA management is to monitor disease activity, which can be used to modify both pharmacological and nonpharmacological treatments [3].

With increased smartphone ownership, mobile health (mHealth) becomes a relevant and fast-growing field of health care delivery, whereby the apps developed for smartphone users could be potential useful tools for both patient self-management and enhanced communications between patients and physicians [4,5]. Apps that targeted the management of chronic diseases had shown benefits for patients in research studies, including conditions such as obesity and diabetes, which led to improved clinical outcomes and maintenance of high-quality medical care [6]. In the field of rheumatology, it had been shown that it was beneficial for patients with rheumatoid arthritis (RA), where management was enhanced with electronic tools such as digitally recorded disease activity (joint counts) and electronic patient-reported outcome measures (ePROMs). These patients with RA who regularly completed ePROMs and digitally tracked their disease had a better compliance to their medications, less activity handicap in daily living, and more positive outlook for their future [7].

There is a need for potential users to be able to objectively evaluate the quality of health care apps as it is unclear for the average user as to which apps offer evidence-based tools and education [6]. It had been shown that many health apps neither adhere to evidence-based guidelines nor involve medical professionals during development [8]. Without a proper evaluation system, users could be trusting apps that are not based on best medical practices or evidence, which could lead to harm. As the availability of such apps increases, it is important that users make educated decisions about the apps they use. For a disease like SpA that has many different subtypes, it has been shown that there are benefits to having a single app that allows patients to monitor their disease activity across all subtypes [2,9].

### Objectives

The objective of this study was to identify existing publicly available, high-quality apps that use validated measurement instruments for monitoring SpA disease activity. The specific aims of this review were to evaluate and determine the features and quality of apps designed to monitor SpA disease activity by (1) identifying and summarizing the available apps and the key disease activity monitoring features, (2) comparing the app features with validated instruments for monitoring of SpA disease activity, and (3) rating app quality according to the Mobile App Rating Scale (MARS) [10]. This will enable users to make informed decisions about specific apps and may identify deficiencies in the mHealth apps for SpA disease activity monitoring currently available for future development purposes.

## Methods

This review comprised 4 stages. Stage 1 involved a systematic search of the apps on both the Apple App Store and Google Play Store based on a combination of keywords (stage 1: app identification). This included a screening process in which apps were screened for inclusion into the next stage. Stage 2 involved identifying the validated disease monitoring instruments for various SpA subtypes. Stage 3 involved the extraction and recording of relevant data regarding each app. Finally, the apps were evaluated (stage 4) using the MARS checklist.

### App Identification

A systematic search of the Apple App Store and Google Play Store was conducted from July 15, 2018, to July 21, 2018, to identify all potentially relevant apps. The review was conducted following the Preferred Reporting Items for Systematic reviews Meta-Analyses guidelines [11]. Search terms included “spondyloarthritis” OR “ankylosing” OR “ankylosing spondylitis” OR “psoriatic” OR “psoriatic arthritis” OR “reactive arthritis” OR “inflammatory bowel disease related arthritis” OR “arthritis.” The app store description of each identified app was read and compared with the inclusion and exclusion criteria. Apps were included if they were (1) a smartphone-based app, (2) capable of running on Android or iOS operation systems, (3) in English language, (4) useful for people with SpA or to assist people with SpA for their clinical care, and (5) available for download in the app store (Apple App Store or Google Play Store). Apps were excluded if (1) a condition other than SpA was targeted; (2) the app included only treatment algorithms; (3) it was explicitly only for clinician use; or (4) app content was for information, education, or reference only (ie, no data entry). Apps not updated before 2017 were also excluded because of potential incompatibility with newer operating systems that could underrepresent the functionality of the app. When an app was found in both the Google Play Store and Apple App Store, both versions were included so any differences between operating systems could be identified, and the reviewers interacted with both apps.

To ensure the capture of all relevant apps, the search on the Apple App Store (using an iPhone on a Singapore internet protocol [IP] address) was compared with a website [12] (Singapore Apple App Store) using the search term “arthritis.” “Arthritis” was used as the search term as it returned the most apps during the main search.

### Validated Spondyloarthritis Disease Activity Monitoring Instruments

As part of our objective was to assess apps that measured disease activity in patients with SpA, these instruments were identified (Table 1) using guidelines from the Assessment of SpondyloArthritis international Society [13] and European League Against Rheumatism [3,14], along with the subtype of SpA they relate to. Apps were then evaluated as to whether they possess the functionality to calculate any of these instruments.

**Table 1:** Validated disease activity monitoring instruments for spondyloarthritis disease activity monitoring.

Instrument	Spondyloarthritis subtype	Instrument components
Bath Ankylosing Spondylitis Functional Index [13]	AS^a^	PGA^b^
Bath Ankylosing Spondylitis Disease Activity Index [13]	AS	PGA
Ankylosing spondylitis disease activity score [3]	AS	PGA, erythrocyte sedimentation rate, and CRP^c^
Minimal disease activity [3]	PsA^d^	28 tender joint count, 28 swollen joint count, VAS^e^, Psoriasis Area Severity Index, body surface area, PGA, Health Assessment Questionnaire, and tender entheseal points
Disease activity index for psoriatic arthritis [3]	PsA	68 tender joint count, 66 swollen joint count, CRP, VAS, and PGA
Psoriatic Arthritis Impact of Disease [14]	PsA	PGA

^a^AS: ankylosing spondylitis.

^b^PGA: patient global assessment (of disease activity).

^c^CRP: C-reactive protein.

^d^PsA: psoriatic arthritis.

^e^VAS: (patient pain) visual analog score.

### Data Extraction

The following data about all apps were recorded: app name, platform (Android or iOS), developer, current version, size, cost, number of installs, and user star ratings. General functions of the apps as well as any validated SpA disease activity monitoring instruments were recorded descriptively.

### App Rating Using the Mobile App Rating Scale

The MARS was developed as a means to determine the quality and classification of mHealth apps [10]. The MARS identified 23 items that were rated on a 5-point scale (1=inadequate, 2=poor, 3=acceptable, 4=good, and 5=excellent), with a description provided for each anchor rating. The MARS comprises 5 different domains and each contributes to the overall evaluation of the app quality:

1. Engagement (5 items)—the extent to which the app engages the target users;
2. Functionality (4 items)—how easy the app is to learn and navigate and overall app performance;
3. Aesthetics (3 items)—the graphics, visual appeal, and style of the app;
4. Information quality (7 items)—the accuracy of the app description, the quality and quantity of information in the app, and whether the information is verified by scientific evidence;
5. Subjective quality (4 items).

Scoring for the MARS was done by calculating a mean for each category and an overall mean score, which has been proven to have good internal consistency and interrater reliability, so it is a reliable method to rate and compare mobile apps [10,15,16]. Apps that score ≥4 out of 5 on the overall MARS rating are considered *good* [17].

All the apps were rated by 2 independent reviewers (MFX and WJO) using the MARS [10]. Before embarking on the review, the 2 reviewers discussed the use of the MARS in the context of apps for people with SpA. The target group was determined to be “all people with SpA aged 18 years or older with some experience using smartphone apps.” The reviewers also considered all items of the MARS and confirmed that all were applicable to SpA and that no additional app-specific items were required [10], as recommended by the developers of the MARS.

Before reviewing all the apps identified in the search, both reviewers assessed and discussed an excluded app to ensure alignment of understanding of the MARS rating criteria. The reviewers then independently rated all apps using the MARS. Each app was used for at least 10 min to gain an adequate understanding of the app functionality. Apps were tested on July 24, 26, and 27, 2018, using an iPhone 8 Plus running on iOS 11.4.1 and an MI NOTE LTE equipped with Android version 6.0. 1 MMB29M, using the app version downloaded on July 24, 2018. Any matters or doubts about specific apps were debated between the 2 reviewers and a third reviewer (YHK) who has significant experience in SpA, and consensus was reached.

Scores were calculated for each MARS item, along with a total mean score. The mean score from 2 reviewers was calculated. No app had been tested in clinical studies. Therefore, item 19 of the MARS, *evidence base*, was excluded from calculations. Interrater reliability of the MARS subscales and total quality score were calculated using the intraclass correlation coefficient (ICC) in STATA SE 14.0 by StataCorp LLC.

## Results

### Overview

**Figure 1:**
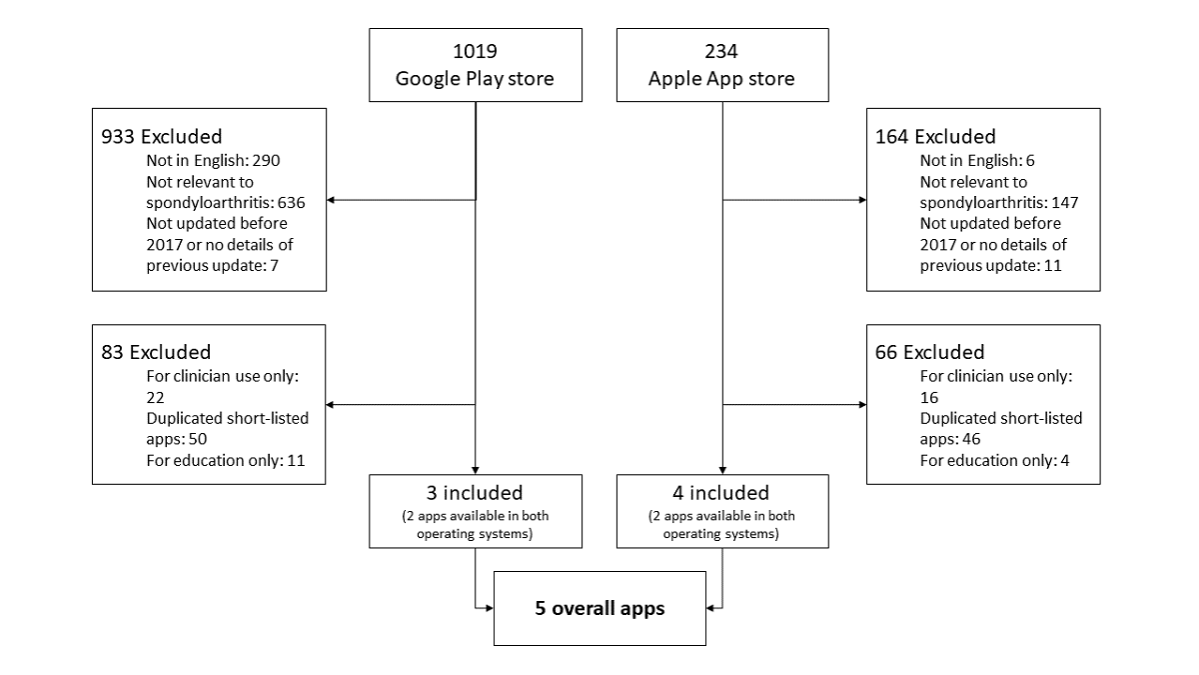
Flow diagram of systematic app search and selection from the Google Play Store and Apple App Store.

The app selection process detailed in the Methods section is summarized in Figure 1. The characteristics and functions of 5 apps were recorded in Tables 2 and 3. The use of validated SpA disease monitoring activity instruments was also recorded, as shown in Table 4. Their MARS scores ranged from 2.93 to 4.04 (possible range 0.00-5.00), as shown in Table 5.

**Table 2:** Operating system, developer, version, and size of included apps.

App	Operating system	Developer	iOS version	iOS size (MB)	Android version	Android size (MB)
AS Health Storylines	iOS and Android	Self Care Catalysts Inc	7.24	18	5.4.4_10MAY2017_1	12
Psoriasis Manager	iOS	At Point of Care LLC	10.0.0	20.7	—^a^	—
GRAPPA^b^ App	iOS and Android	GRAPPA	1.1	30.3	1.1	26
ArthritisPower	iOS	Global Healthy Living Foundation Inc	2.0.6	14.9	—	—
MySpA	Android	Barts Health National Health Service Trust	—	—	1.1.6	80

^a^Not available.

^b^GRAPPA: Group for Research and Assessment of Psoriasis and Psoriatic Arthritis.

**Table 3:** App description and target users.

App	App description	Target subtype (as per app description)
AS Health Storylines	Input data to monitor disease	Ankylosing spondylitis
Psoriasis Manager	Input data to monitor disease	PsA^a^
Group for Research and Assessment of Psoriasis and Psoriatic Arthritis App	Input data to monitor disease	PsA
ArthritisPower	Input data to monitor disease	PsA
MySpA	General information; exercise plans; input data to monitor disease	Axial spondyloarthritis and PsA

^a^PsA: psoriatic arthritis.

**Table 4:** App inclusion of the various spondyloarthritis activity measure and component measurement instruments and other functionality of included apps.

App	Data entered	Instruments or laboratory measures	Composite disease activity measure	Allows users to record and retrieve disease activity data on multiple occasions	General functions
AS Health Storylines	Medication and symptoms	PGA^a^	—^b^	History (journal style) and graph (trend of scores)	Symptom and medication tracker
Psoriasis Manager	Medication and symptoms	PGA	—	History (journal style)	Treatment tracker and patient journal
Group for Research and Assessment of Psoriasis and Psoriatic Arthritis App	Symptoms	PGA	Psoriatic Arthritis Impact of Disease and minimal disease activity	—	Disease monitoring information about PsA^c^
ArthritisPower	Medication, symptoms, and laboratory results	PGA	—	History (journal style) and graph (trend of scores)	Symptom and medication tracker
MySpA	Medication, symptoms, and laboratory results	PGA, 66/68 joint count, C-reactive protein, and erythrocyte sedimentation rate	Bath Ankylosing Spondylitis Functional Index and Bath Ankylosing Spondylitis Disease Activity Index	History (journal style)	Information about axial spondyloarthritis and PsA disease monitoring

^a^PGA: patient global assessment (of disease activity).

^b^Not available.

^c^PsA: psoriatic arthritis.

**Table 5:** Mean Mobile App Rating Scale ratings of included apps.

App name	MARS^a^ categories											
	Engagement mean scores (5 items)		Functionality mean scores (4 items)		Aesthetics mean scores (3 items)		Information mean scores (7 items)		Subjective mean scores (4 items)		Overall MARS mean scores	
	Android	iOS	Android	iOS	Android	iOS	Android	iOS	Android	iOS	Android	iOS
AS Health Storylines	2.00	2.00	3.75	3.75	3.67	3.67	3.00	3.00	2.25	2.25	2.93	2.93
Psoriasis Manager	—^b^	2.80	—	4.00	—	3.33	—	3.00	—	2.25	—	3.08
Group for Research and Assessment of Psoriasis and Psoriatic Arthritis App	3.40	3.40	4.50	4.50	4.33	4.33	4.20	4.20	3.75	3.75	4.04	4.04
ArthritisPower	—	2.40	—	4.00	—	3.33	—	3.50	—	2.00	—	3.05
MySpA	3.40	—	4.50	—	4.33	—	4.20	—	3.75	—	4.04	—

^a^MARS: Mobile App Rating Scale.

^b^Not available.

The search retrieved 1019 Android apps from the Google Play Store. Of these, 1016 were excluded, leaving 3 apps for analysis (Figure 1). A total of 234 iOS apps were retrieved from the Apple App Store. After exclusion of 230 apps, 4 apps remained for analysis. A total of 91 apps were found in the concurrent fnd.io search of the Singapore Apple App Store, out of which no relevant further apps were found. As 2 apps were available in both operating systems, a total of 5 different apps were included; all were free apps.

### Characteristics and Functions of Included Apps

The information on app platform, developer, version, and size are shown in Table 2. As no app had different function between operating systems, the apps are presented only once in Tables 2-5. The app description and target user (as derived from the app store description) are shown in Table 3. None of the iOS apps included had any star rating. Table 4 shows data entry and main functionality in the apps. All the apps allowed users to enter data, such as symptoms and medication. One app (MySpA) included the Bath Ankylosing Spondylitis Disease Activity Index (BASDAI) and Bath Ankylosing Spondylitis Functional Index (BASFI) measures, whereas another app (Group for Research and Assessment of Psoriasis and Psoriatic Arthritis [GRAPPA] App) included the Psoriatic Arthritis Impact Of Disease (PsAID) measures and the minimal disease activity (MDA) calculator with the Health Assessment Questionnaire (HAQ) and Psoriasis Area and Severity Index embedded within.

App inclusion of component measurement instruments, composite disease activity measures calculated, and app functionality to record and retrieve data over time are shown in Table 4. A total of 2 apps (GRAPPA App and MySpA) included at least one validated composite measure of SpA disease activity, out of which none provided the formulae for calculation of the composite disease activity measure. Moreover, 4 apps (AS Health Storylines, Psoriasis Manager, ArthritisPower, and MySpA) included a function allowing data (such as patients’ medication and symptoms) to be recorded and retrieved. In addition, 1 app (MySpA) included both composite disease activity measure and allowed data recording and retrieval.

### Rating of Apps on Mobile App Rating Scale

The MARS ratings for included apps are shown in Table 5. The ICC for the MARS ratings were greater than or equal to 0.91 for all the MARS sections. For overall MARS ratings, the ICC was 0.99 (95% CI 0.98-0.99), confirming good interrater reliability. Of the 2 apps that scored ≥4 out of 5 on the overall MARS rating (considered good [17]), both apps (MySpA and GRAPPA App) included composite disease activity measures that were validated (BASFI and BASDAI for MySpA; MDA and PsAID for GRAPPA App), but only MySpA had a data tracking function. The overall MARS scores for the apps ranged from 2.93 to 4.04, indicating significant variation in the quality of apps. Subjective quality (2.00-3.75) and engagement (2.00-3.40) showed greatest variability.

## Discussion

### Principal Findings

The aim of this study was to identify existing high-quality apps that used validated measurement instruments for monitoring SpA disease activity. This review of apps showed that a significant proportion of the publicly available apps specifically designed for SpA were for general education purposes only (50% [4/8] for iOS apps and 78% [11/14] for Android apps), whereas there was a smaller category of apps for tracking SpA symptoms and calculation of validated disease activity measures (50% [4/8] for iOS apps and 22% [3/14] for Android apps). Of the symptom-tracking apps, 60% (3/5) did not use validated instruments in this study. We would recommend patients to use apps with validated disease activity tracking instruments and app developers to develop future apps using such instruments as well.

Only 1 app (scoring ≥4 out of 5 on the overall MARS), MySpA, included both a symptom-tracking function and calculation of validated composite measures of AS disease activity (BASFI and BASDAI). Hence, patients with AS wishing to track their symptoms are encouraged to use MySpA. Another app, GRAPPA App (also scoring ≥4 out of 5 on the overall MARS), included the calculation of validated composite measures of PsA disease activity (MDA and PsAID) but lacked a tracking function. People with PsA can opt to use the app for calculating their disease activity scores but may want to record it elsewhere for tracking purposes. Although the app AS Health Storylines does not have the function to calculate a validated disease activity monitoring score, it has the function to track and remind patients to take medications, which can potentially be synchronized with the overall health care ecosystem. App developers can consider adding both the calculation and recording functions to future developments.

Although some disease activity instruments can be calculated easily by patients (via apps) using ePROMs, such as the BASDAI, BASFI, and PsAID, there are others that require either clinician inputs (tender and swollen joint counts and tender entheseal points for MDA) or laboratory test results (C-reactive protein and erythrocyte sedimentation rate for the Ankylosing Spondylitis Disease Activity Score). There are studies showing that self-reported joint counts have shown to reasonably predict clinician-performed joint counts for RA patients [18,19], though this assumption that self-reported joint counts will be sufficient for measurement of SpA disease activity needs to be validated further. GRAPPA App, which has a calculator for MDA, includes an HAQ questionnaire that the patient completes him or herself. It is also worth noting that no app included tracking of disease activity for more than 1 SpA subtype and that apps for tracking disease activity of SpA subtypes other than AS and PsA were not found. There is potential for future app developers to consider filling in these gaps or developing a single app with the capability of disease monitoring across all the different subtypes of SpA. We would also like to highlight that although these apps are useful in helping the patients track their disease activity, appropriate treatment and therapy should still be done in collaboration with their health care teams.

Apps rated have a wide range of the MARS scores (2.93 to 4.04), indicating highly inconsistent quality of apps in terms of user experience. App developers wishing to optimize user experience can consider using the MARS criteria as a checklist, along with collaborating with the key stakeholders such as people with SpA and medical professionals. Item 19 of the MARS, *evidence base*, was excluded from all calculations because no app had been studied in clinical trials [10]. Therefore, clinical trials should be conducted for any future apps developed for SpA disease activity monitoring to determine the clinical impact on outcomes for people with SpA, as well as to ensure cost-effectiveness and to undergo external quality review [20].

This review has limitations. Only apps available in app stores accessed from a Singapore IP address and in English language were included. However, a preliminary search of the iTunes store of the United States with the term “arthritis” suggested that the main search of the app stores had captured all relevant apps in English language. Patients were not included in the rating of the app in this study. Future studies can be performed to address this gap.

App quality was assessed using the MARS. Although the MARS was recently developed and had not been extensively validated, it had now been used in several other app evaluations [15,16,21], and had consistently proven good interrater reliability between reviewers. The ICC was 0.99 for the overall MARS score in this study, confirming good interrater reliability. Caregivers can consider using the MARS criteria for rating apps that they wish to recommend to their patients.

Assessment of data privacy and security was not included in the MARS but was a commonly considered criterion of health software quality not included in this study [22]. Data privacy and security considerations are of utmost importance and will need to be evaluated against the specific regulatory requirements of the country in which the app is being used and should be included in future studies.

### Conclusions

In conclusion, to our knowledge, this is the first review of high-quality apps for monitoring SpA disease activity that use validated measurement instruments. This review indicated that the apps MySpA and GRAPPA App were high-quality apps that used validated disease activity measures, which could assist in the management of SpA subtypes AS and PsA, respectively. However, there is a lack of apps that consolidate the measurement and tracking of disease activity of different SpA subtypes into a single app. Future app development can consider developing apps that bridge these gaps.

## Abbreviations

AS: ankylosing spondylitis
BASDAI: Bath Ankylosing Spondylitis Disease Activity Index
BASFI: Bath Ankylosing Spondylitis Functional Index
CRP: C-reactive protein
ePROM: electronic patient-reported outcome measure
GRAPPA: Group for Research and Assessment of Psoriasis and Psoriatic Arthritis
HAQ: Health Assessment Questionnaire
ICC: intraclass correlation coefficient
IP: internet protocol
MARS: Mobile App Rating Scale
MDA: minimal disease activity
mHealth: mobile health
PGA: patient global assessment
PsA: psoriatic arthritis
PsAID: Psoriatic Arthritis Impact of Disease
RA: rheumatoid arthritis
SpA: spondyloarthritis
VAS: visual analog score

## References

[1] Raychaudhuri SP, Deodhar A (2014). The classification and diagnostic criteria of ankylosing spondylitis. J Autoimmun.

[2] Png K, Kwan YH, Leung YY, Phang JK, Lau JQ, Lim KK, Chew EH, Low LL, Tan CS, Thumboo J, Fong W, Østbye T (2018). Measurement properties of patient reported outcome measures for spondyloarthritis: a systematic review. Semin Arthritis Rheum.

[3] Smolen JS, Schöls M, Braun J, Dougados M, FitzGerald O, Gladman DD, Kavanaugh A, Landewé R, Mease P, Sieper J, Stamm T, Wit MD, Aletaha D, Baraliakos X, Betteridge N, Bosch FV, Coates LC, Emery P, Gensler LS, Gossec L, Helliwell P, Jongkees M, Kvien TK, Inman RD, McInnes IB, Maccarone M, Machado PM, Molto A, Ogdie A, Poddubnyy D, Ritchlin C, Rudwaleit M, Tanew A, Thio B, Veale D, Vlam KD, van der Heijde D (2018). Treating axial spondyloarthritis and peripheral spondyloarthritis, especially psoriatic arthritis, to target: 2017 update of recommendations by an international task force. Ann Rheum Dis.

[4] World Health Organization (2011). mHealth: New Horizons for Health Through Mobile Technologies - Second Global Survey on Ehealth.

[5] Becker S, Miron-Shatz T, Schumacher N, Krocza J, Diamantidis C, Albrecht UV (2014). mHealth 2.0: experiences, possibilities, and perspectives. JMIR Mhealth Uhealth.

[6] Luo D, Wang P, Lu F, Elias J, Sparks JA, Lee YC (2019). Mobile apps for individuals with rheumatoid arthritis: a systematic review. J Clin Rheumatol.

[7] El Miedany Y, El Gaafary M, Youssef S, Bahlas S, Almedany S, Ahmed I, Palmer D (2016). Toward electronic health recording: evaluation of electronic patient-reported outcome measures system for remote monitoring of early rheumatoid arthritis. J Rheumatol.

[8] Subhi Y, Bube SH, Bojsen SR, Thomsen AS, Konge L (2015). Expert involvement and adherence to medical evidence in medical mobile phone apps: a systematic review. JMIR Mhealth Uhealth.

[9] Kwan YH, Fong W, Tan VI, Lui NL, Malhotra R, Østbye T, Thumboo J (2017). A systematic review of quality-of-life domains and items relevant to patients with spondyloarthritis. Semin Arthritis Rheum.

[10] Stoyanov SR, Hides L, Kavanagh DJ, Zelenko O, Tjondronegoro D, Mani M (2015). Mobile app rating scale: a new tool for assessing the quality of health mobile apps. JMIR Mhealth Uhealth.

[11] Moher D, Liberati A, Tetzlaff J, Altman DG, PRISMA Group (2009). Preferred reporting items for systematic reviews and meta-analyses: the PRISMA statement. PLoS Med.

[12] Fnd: Experience the App Store and iTunes Anywhere.

[13] Sieper J, Rudwaleit M, Baraliakos X, Brandt J, Braun J, Burgos-Vargas R, Dougados M, Hermann K, Landewé R, Maksymowych W, van der Heijde D (2009). The assessment of spondyloarthritis international society (ASAS) handbook: a guide to assess spondyloarthritis. Ann Rheum Dis.

[14] Gossec L, de Wit M, Kiltz U, Braun J, Kalyoncu U, Scrivo R, Maccarone M, Carton L, Otsa K, Sooäär I, Heiberg T, Bertheussen H, Cañete JD, Lombarte AS, Balanescu A, Dinte A, de Vlam K, Smolen JS, Stamm T, Niedermayer D, Békés G, Veale D, Helliwell P, Parkinson A, Luger T, Kvien TK, EULAR PsAID Taskforce (2014). A patient-derived and patient-reported outcome measure for assessing psoriatic arthritis: elaboration and preliminary validation of the psoriatic arthritis impact of disease (PsAID) questionnaire, a 13-country EULAR initiative. Ann Rheum Dis.

[15] Grainger R, Townsley H, White B, Langlotz T, Taylor WJ (2017). Apps for people with rheumatoid arthritis to monitor their disease activity: a review of apps for best practice and quality. JMIR Mhealth Uhealth.

[16] Mani M, Kavanagh DJ, Hides L, Stoyanov SR (2015). Review and evaluation of mindfulness-based iPhone apps. JMIR Mhealth Uhealth.

[17] Reyes A, Qin P, Brown CA (2018). A standardized review of smartphone applications to promote balance for older adults. Disabil Rehabil.

[18] Pincus T, Yazici Y, Bergman M, Maclean R, Harrington T (2007). A proposed continuous quality improvement approach to assessment and management of patients with rheumatoid arthritis without formal joint counts, based on quantitative routine assessment of patient index data (RAPID) scores on a multidimensional health assessment questionnaire (MDHAQ). Best Pract Res Clin Rheumatol.

[19] Cheung PP, Gossec L, Mak A, March L (2014). Reliability of joint count assessment in rheumatoid arthritis: a systematic literature review. Semin Arthritis Rheum.

[20] Buijink AW, Visser BJ, Marshall L (2013). Medical apps for smartphones: lack of evidence undermines quality and safety. Evid Based Med.

[21] Patel R, Sulzberger L, Li G, Mair J, Morley H, Shing MN, O'Leary C, Prakash A, Robilliard N, Rutherford M, Sharpe C, Shie C, Sritharan L, Turnbull J, Whyte I, Yu H, Cleghorn C, Leung W, Wilson N (2015). Smartphone apps for weight loss and smoking cessation: quality ranking of 120 apps. N Z Med J.

[22] Lewis TL, Wyatt JC (2014). mHealth and mobile medical apps: a framework to assess risk and promote safer use. J Med Internet Res.

